# Measuring Experiential Avoidance: Evidence toward Multidimensional Predictors of Trauma Sequelae

**DOI:** 10.3390/bs7010009

**Published:** 2017-02-20

**Authors:** Meaghan Lewis, Amy Naugle

**Affiliations:** Department of Psychology, Western Michigan University, 3700 Wood Hall, Kalamazoo, MI 49008, USA; amy.naugle@wmich.edu

**Keywords:** experiential avoidance, MEAQ, AAQ-II, childhood trauma

## Abstract

The current study sought to investigate measurement discrepancies in self-report assessment of experiential avoidance (EA). Recent research indicates that EA may be more appropriately conceptualized as a multidimensional construct, operationally defined in terms of specific avoidance strategies. To test this notion, EA was measured using two self-report assessment instruments, the Acceptance and Action Questionnaire-II (AAQ-II) and the Multidimensional Experiential Avoidance Questionnaire (MEAQ) in a convenience sample of university students. Measurement differences across measures and unique contributions to prediction of posttraumatic stress symptoms (PTSS) and engagement in problematic behaviors were evaluated. Both the AAQ-II and MEAQ were found to significantly mediate the effect of childhood trauma exposure on PTSS. However, when levels of PTSS were dummy coded into dichotomies of those with a likely PTSD diagnosis and those without, the MEAQ was a stronger predictor of symptoms for those with a likely PTSD diagnosis than the AAQ-II. These results provide initial support for the discriminant validity of the MEAQ, which appears to be a more specific predictor of trauma-related symptoms.

## 1. Introduction

While self-report methodology is not an optimal mode of research, researchers interested in capturing private events (e.g., thoughts, feelings, memories) have historically used such methodology due to the limited accessibility of such events. Participants in psychology research studies are frequently instructed to endorse statements on Likert-type scales in order to report on private experiences that would otherwise be unknown to outside observers. One common construct of the ways in which individuals attempt to ameliorate contact with unwanted private experiences is described as experiential avoidance (EA). EA is a construct first outlined in the Acceptance and Commitment Therapy (ACT) literature defined as: (a) an unwilling stance toward private events; and (b) involving operant escape or avoidance of aversive private events [1]. The process of EA is conceptualized as being sustained through negative reinforcement. In the ACT literature, EA is considered a transdiagnostic process that transcends psychological suffering regardless of psychiatric diagnoses [2]. That is, the form of mental illness matters less than the underlying function of the behavior thought to maintain one’s suffering. 

Although the process of EA is thought to maintain many psychiatric illnesses, not all instances of avoidance are considered pathogenic. Problematic instances are rather defined in terms of the extent to which engaging in avoidant responding limits one’s contact with meaningful domains, or one’s values (e.g., education, family, spirituality) [3]. When EA is employed chronically, it may become a pervasive pattern of negative reinforcement. Use of such a strategy thus conceivably limits contact with direct reinforcement contingencies that would otherwise facilitate new learning. That is, chronic avoidance of unwanted private events limits valuable learning opportunities that one’s world is not, in fact, dangerous. The panic-disordered individual, for example, may avoid contact with stimuli that trigger interoceptive arousal. Thereby, one fails to learn that such cues do not equate to catastrophic outcomes. Researchers posit that EA then seems to be a generalized psychological vulnerability for a host of psychological symptoms and behavioral problems [4]. In this line of thinking, engaging in EA may help explain psychological vulnerabilities to various trajectories of mental illness that, while different in form, can be explained by a unifying avoidance function. 

This unifying avoidance function may be partially maintained by thought suppression. Research evidence supports the notion that thought suppression or distraction from one’s own covert verbal behavior appears to result in a paradoxical reemergence of those same events despite best efforts to avoid [5]. Relatedly, the empirical associations between EA and a number of harmful psychological and behavioral problems such as substance and alcohol misuse, post-traumatic stress disorder (PTSD), depression, and anxiety [6,7,8] support this premise. Engaging in pervasive EA may result in short-term relief, but long-term harm as the aforementioned consequences appear related to higher levels of EA. Another research group found that multidimensional components of EA explained the relationship between comorbid PTSD and alcohol use disorders [9]. In this vein, different avoidance strategies (e.g., behavioral avoidance or distraction and suppression) could be potent avoidance strategies that ultimately maintain comorbidities such as PTSD and alcohol use disorders. Existing definitions of EA hold that it is perhaps most accurately conceptualized as a functional response class [10]. Thus, it would be inaccurate to define EA solely in terms of a formal unit of analysis (e.g., defining EA by the way it appears rather than the underlying function it serves). Instead, patterns of behavior are conceptualized as representing EA to the extent that their escape or avoidance function limit one’s contact with valued activities. 

Trauma exposure is one area in which strategies used to manage private events seems to substantially predict long-term outcomes [11,12,13,14,15]. Adopting an unwilling stance toward experiencing emotions post-trauma (e.g., engaging in various forms of EA) is presumed to predict harmful consequences such as drug and alcohol misuse and risky sexual behavior, and further impair use of helpful emotion regulation strategies [16,17,18]. In particular, trauma exposure during childhood may interfere with the development of healthy emotion regulation strategies and increase the future probability of engaging in EA in similar contexts in the future [19]. To illustrate, EA was found to mediate the relationship between childhood trauma exposure and later psychological symptoms in adulthood in several samples [20,21]. This research seems to bolster the conceptualization of EA as a vulnerability factor for harmful long-term outcomes. Childhood trauma exposure itself may not be the only critical factor in predicting suffering in adulthood. Rather, the emotion regulation strategies (e.g., suppression) developing post childhood trauma exposure, while producing initial symptom reduction, may result in later harm. 

If EA is to be conceptualized as a functional response class as opposed to hypothetical construct, it should also hold that a multitude of problematic behaviors serve an EA function. In support of this hypothesis, it was found that EA explained co-varying relationships between ten different problematic behaviors such as sexual promiscuity, substance/alcohol misuse, internet overuse, and binge/restrictive eating [22]. Similarly, alcohol use was established as a predictor of internet overuse by a different research group [23]. Given the evidence for relationships between various excessive behaviors that differ in topography, a functional approach may be more suited to account for their relationships. As such, individuals who engage in EA at an increased rate may have a heightened risk of engaging in problematic behaviors. This point may be particularly true when contextual cues evoke distressing private events, thus signaling avoidance behavior. 

While researchers are making strides toward developing behavioral methods of measuring EA, as mentioned above, self-report remains the most commonly used strategy for measuring this construct. The primary self-report instrument to measure EA is currently the Acceptance and Action Questionnaire-II (AAQ-II) [24]. Given problems related to discriminant validity from similar constructs such as neuroticism, the Multidimensional Experiential Avoidance Questionnaire (MEAQ) was developed [1] with an aim to improve both the psychometric properties and operational definition of EA. Specific behaviors that seem to load onto EA (e.g., drug use and internet overuse) were elucidated in the final item pool. The MEAQ measures specific avoidance strategies such as distress aversion and distraction or suppression that might be employed to escape or avoid aversive private events. The authors report stronger psychometric properties and assessment of broader content related to EA. 

The purpose of the present study was to methodologically examine the predictive validity of the AAQ-II and the MEAQ in relation to posttraumatic stress symptoms (PTSS) and problematic behaviors. Comparisons between the AAQ-II and MEAQ in their relative contributions to predicting a likely PTSD diagnosis were made. Based on the psychometric and conceptual differences in the operational definition of EA and evidence of differences in the AAQ-II and MEAQ’s prediction of PTSS in an earlier study [25], it was hypothesized that measurement differences would be found between the AAQ-II and MEAQ in their prediction of PTSS and problematic behaviors. Given the MEAQ’s item content involving specific avoidance strategies that conceptually map onto the EA construct, it was hypothesized that the MEAQ would offer incremental specificity above and beyond the AAQ-II in predicting PTSS and problematic behaviors. Mediational hypotheses were developed based on research evidence supporting indirect effects of EA on trauma outcomes, including alcohol use and trauma symptom severity in a convenience sample [9]. It was hypothesized that participants’ responses to the AAQ-II would significantly mediate the effect of childhood trauma exposure on PTSS. It was also hypothesized that MEAQ scores would significantly mediate the relationship between childhood trauma exposure and PTSS. Differences between the AAQ-II and MEAQ’s relationships to PTSS and problematic behaviors were expected to emerge when groups were divided into likely PTSD and unlikely PTSD diagnosis categories. This particular hypothesis was formulated on the AAQ-II’s psychometric criticism [1] related to poor discriminant validity. 

## 2. Materials and Methods

### 2.1. Participants

Participants consisted of 226 undergraduate students recruited from a medium sized Midwestern university. The mean age was 20 (*SD* = 9.16), 77% described themselves as female, and 71% identified as white or Caucasian. All study procedures received human subjects’ institutional review before implementation. Participants were recruited from university classrooms by trained research assistants who read from a script detailing the study, explaining that the survey would ask questions related to emotional responses to trauma. Self-report measures were completed online using the Survey Monkey website. Extra credit was provided based on instructor discretion. No other forms of compensation for participation were provided. 

### 2.2. Measures

Experiential avoidance was measured using two well-validated self-report measures. The AAQ-II [24] is a 7-item self-report measure of EA and general psychological inflexibility. The AAQ-II is the most widely used measure of EA to date. Items are responded to on seven-point Likert-type scale (ranging from 1 = *never true* to 7 = *always true*). Higher scores are indicative of greater EA, while lower scores reflect increased psychological flexibility. The AAQ-II has demonstrated good internal consistency (alpha coefficient mean of 0.84). It also has good test-retest reliability of 0.81 and 0.79 for twelve and three months, respectively. Internal consistency of the AAQ-II in the present study was good (α = 0.89).

The MEAQ [1] is a 62-item self-report measure that was developed to address a wider range of EA than captured by the AAQ and AAQ-II. It was also developed to address issues with internal consistency and poor discriminant validity that have been evidenced with other measures of EA. The MEAQ contains questions pertaining to six dimensions of EA: behavioral avoidance, distress aversion, procrastination, distraction and suppression, repression and denial, and distress endurance. Items are rated on a Likert-type scale, ranging from 1 = *strongly disagree* to 6 = *strongly agree,* and higher scores are indicative of greater EA. The MEAQ has demonstrated good internal consistency and excellent convergent validity with avoidance measures and related constructs including thought suppression, stress avoidance, social avoidance, and alexithymia. It also has excellent discriminative validity and greater assessment of unique content through the six subscales. The internal consistency for the MEAQ in the present study was good (α = 0.87). The MEAQ subscales also demonstrated good internal consistency, behavioral avoidance (α = 0.80); distress aversion (α = 0.81); procrastination (α = 0.80); distraction and suppression (α = 0.80); repression and denial (α = 0.82); and distress endurance (α = 0.81). 

The Childhood Trauma Questionnaire – Short Form (CTQ-SF) [26] is a 28-item measure intended to assess traumatic experiences in childhood, before the age of 17. Items are endorsed on a 5-point rating scale (ranging from 1 = *never true* to 5 = *very often true*). Subscales include emotional abuse, physical abuse, sexual abuse, emotional neglect, and physical neglect. The CTQ-SF also includes a three item validity scale that assesses minimization/denial to capture potential underreporting of child maltreatment. Bernstein et al. (2003) [26] report good criterion-related validity across four sample pools including substance abusing patients (*n* = 378), adolescent psychiatric inpatients (*n* = 396), substance abusing individuals recruited from southwest Texas (*n* = 625), and a normative community sample (*n* = 579), total *N* = 1978. Internal consistency of the CTQ-SF in the present study was good (α = 0.87). 

PTSS were assessed using the Posttraumatic Stress Disorder Checklist-Civilian Version (PCL-C) -5 [27]. The PCL-5 is a 20-item self-report assessment instrument developed to assess DSM-5 PTSD symptomatology based on the criteria changes with the introduction of the DSM-5. Internal consistency was excellent in a sample of trauma-exposed college students (α = 0.94). The PCL-5 also has good test-retest reliability (*r* = 0.82) and evidences convergent (*r*s = 0.74 to 0.85) and discriminant validity (*r*s = 0.31 to 0.60) [28]. The PCL-5’s internal consistency in the present study was excellent (α = 0.93). Thirty-four percent of participants fell at or above the recommended cut-score [28]. 

Engagement in problematic behaviors was assessed using the Composite Measure of Problem Behaviors (CMPB) [29]. The CMPB is a 46-item measure of ten different problems behaviors including: nicotine use, deliberate self-harm, excessive internet/computer game use, drug use, excessive exercise, excessive alcohol use, binge eating, sexual promiscuity, aggression, and restrictive eating. Participants endorse the items on a six-point scale (ranging from 1 = *very unlike me* to 6 = *very like me*). Confirmatory factor analysis delineated a common higher order factor explaining covariance between the subscales and was developed and validated based on the common finding that problem behaviors co-occur. The subscales of the CMPB have good construct validity with other psychometrically validated measures including the Alcohol Use Disorders Identification Test (*r* = 0.76), the Sociosexual Orientation Inventory (*r* = 0.56), the Three Factor Eating Questionnaire (*r* = 0.50), and the Deliberate Self-Harm Inventory (*r* = 0.71; see [29]. The CMPB also has good internal consistency (α = 0.73–0.91) and test-retest reliability (95% CI). Reliability estimates were also stable across time periods of: two weeks (*r* = 0.73–0.98), two-four months (*r* = 0.69–0.91), and eight-fourteen months (*r* = 0.65–0.91). Internal consistency in the present study was good (α = 0.80).

### 2.3. Analytic Strategy

All data analyses were conducted using Statistical Packaging for the Social Sciences (SPSS) Software version 21 (IBM, Armonk, NY, USA). Process software [30] was downloaded into SPSS and used to conduct mediational analyses. Skewness was assessed through evaluating the absolute values and graphically through examining the shape of the distribution of study variables. Skewness values that have coefficient values between −1 and +1 are considered normal [31]. To correct for positive skewness observed in childhood trauma history (skewness = 1.98, *SE* = 0.16) and PTSS (skewness = 1.18, *SE* = 0.16), log transformations were computed which reduced the skewness values below the value of one. Missing data were at an acceptable level (missing value percentage = 13%, with 120 of 226 cases missing values) to use the multiple imputation approach [32] and thus this approach was pursued. Patterns of missing data were first evaluated and all missing values were determined to be missing completely at random (MCAR). The maximum-likelihood estimation imputation of missing values approach was therefore employed to impute the missing values. All analyses were first run with the inclusion of the avoidance items in the PCL-5 and then replicated without these items to test whether the overlapping variance in PTSD avoidance and EA was responsible for statistical effects. No significant differences in the analyses were noted and as such, analyses are reported on based on those inclusive of the PCL-5 avoidance items.

## 3. Results

### 3.1. Bivariate Correlations

Pearson’s product moment correlation analyses were used to elucidate the direction and degree of the relationship between the study variables. The results of the analyses and those that follow are based on pooled estimates generated using the multiple imputation approach. EA per the MEAQ was moderately correlated with PTSS, *r* = 0.39 (*p* < 0.01), while EA per the AAQ-II was strongly associated with PTSS, *r* = 0.62 (*p* < 0.01). MEAQ and AAQ-II scores were moderately associated with problematic behaviors, *r* = 0.29 and .32 (*p* < 0.01), respectively. Childhood trauma history was moderately correlated with PTSS, *r* = 0.40 (*p* < 0.01) and problematic behaviors, *r* = 0.28 (*p* < 0.01). Problematic behaviors were moderately associated with PTSS, *r* = 0.37 (*p* < 0.01). Correlations are displayed in Table 1.

### 3.2. Non-Parametric Bootstrapping Analyses

Several non-parametric bootstrapping multiple mediation models were estimated using Process downloadable software in SPSS (IBM, Armonk, NY, USA) by Hayes [30]. Non-parametric bootstrapping does not assume normality as Baron and Kenny’s [33] regression test requires, and demonstrates greater power in detecting mediation over Sobel testing. As such, the bootstrapping method was selected. These multiple mediation models were estimated to test the indirect effects of AAQ-II and MEAQ scores as mediators on the association between childhood trauma history and PTSS and problematic behaviors as the respective outcomes for each model. 

**Figure 1:**
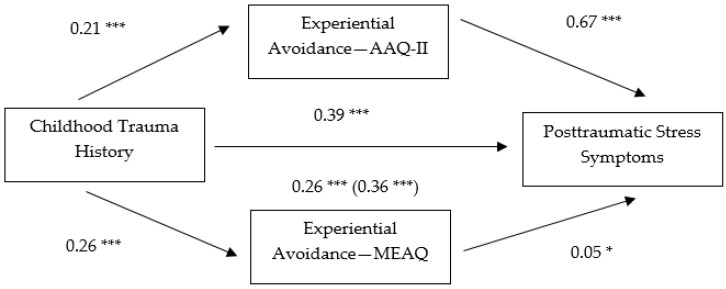
Indirect effect of experiential avoidance (AAQ-II and MEAQ) on the relationship between childhood trauma history and PTSS. Note: *p < 0.05, **p < 0.01, *** *p* < 0.001. Path values were drawn using unstandardized regression coefficients. Parentheses indicate the total effect.

In the first multiple mediation model, the indirect effect of the AAQ-II and the MEAQ on the relationship between the predictor variable, childhood trauma history and PTSS as the outcome variable was estimated. One thousand bootstrap samples were computed with BCa confidence intervals to analyze the indirect effects. Confidence interval values may not contain zero if a mediation effect is to be inferred. Childhood trauma history demonstrated a significant direct effect on both AAQ-II and MEAQ scores and AAQ-II and MEAQ scores showed a significant direct effect on PTSS. AAQ-II scores had a significant indirect effect on the relationship between childhood trauma history and PTSS, 95% BCa CI [0.0925, 0.2392]. MEAQ scores also showed a significant indirect effect on this relationship, 95% BCa CI [−0.0001, 0.0624]. The direct effect in both cases was still significant after analyzing the total effect, which suggests a partial mediating effect of AAQ-II and MEAQ scores on the relationship between childhood trauma history and PTSS. The total effect was significant, 95% BCa CI [0.1090, 0.2504]. Figure 1 displays the indirect effects. 

**Figure 2:**
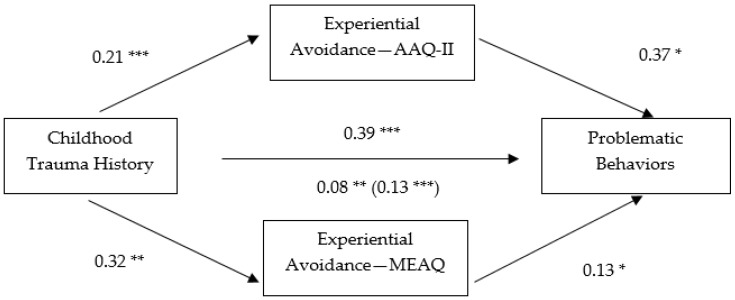
Indirect effect of experiential avoidance (AAQ-II and MEAQ) on the relationship between childhood trauma history and problematic behaviors. Note: * *p* < 0.05, ** *p* < 0.01, *** *p* < 0.001. Path values were drawn using unstandardized regression coefficients. Parentheses indicate the total effect.

Next, a multiple mediation model was computed to test the indirect effect of the AAQ-II and MEAQ on the link between childhood trauma history and engagement in problematic behaviors. Childhood trauma history was noted to have a significant direct effect on AAQ-II and MEAQ scores and AAQ-II and MEAQ scores had a significant direct effect on engagement in problematic behaviors. Results of this analytic strategy revealed AAQ-II scores to have a significant indirect effect on the association between childhood trauma history and problematic behaviors, 95% BCa CI [−0.0062, 0.1962]. MEAQ scores were also observed to have a significant indirect effect on this association, 95% BCa CI [0.0053, 0.1198]. The direct effect remained significant but was reduced. As such, it was concluded the AAQ-II and MEAQ scores partially mediated the effect of childhood trauma history on engagement in problematic behaviors. The total effect was noted to be significant, 95% BCa CI [0.0368, 0.2358]. The indirect effects are on display in Figure 2. 

### 3.3. Likely PTSD Diagnosis

**Figure 3:**
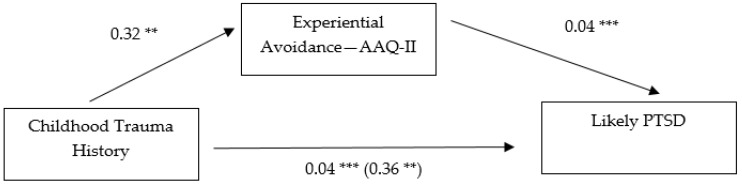
Indirect effect of experiential avoidance (AAQ-II) on the relationship between childhood trauma history and a likely PTSD diagnosis. Note: * *p* < 0.05, ** *p* < 0.01, *** *p* < 0.001. Path values were drawn using unstandardized regression coefficients. Parentheses indicate the total effect.

**Figure 4:**
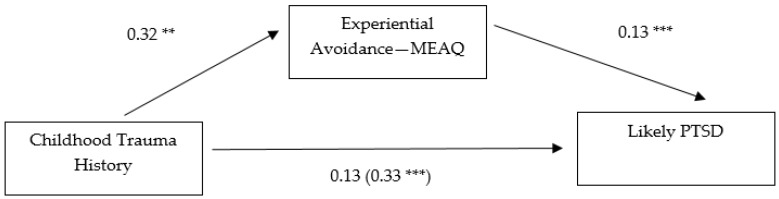
Indirect effect of experiential avoidance (MEAQ) on the relationship between childhood trauma history and a likely PTSD diagnosis. Note: * *p* < 0.05, ** *p* < 0.01, *** *p* < 0.001. Path values were drawn using unstandardized regression coefficients. Parentheses indicate the total effect.

Next, analyses were pursued to make group comparisons among participants with and without a likely PTSD diagnosis. A binary dependent variable was created to dichotomize participants whose PCL-5 scores were at or above the suggested cut-score of 33 and participants whose PCL-5 scores were 32 or below. For comparison purposes, two single mediation models were computed to predict the binary dependent variable from childhood trauma history, AAQ-II scores, and MEAQ scores with positive associations indicative of a stronger relationship to a likely PTSD diagnosis. Thirty-four percent of the sample (*n* = 78) fell at or above the cut-score of 33 for a likely PTSD diagnosis, while 66% of participants (*n* = 148) fell below the cut-score. Childhood trauma history served as a significant direct predictor of AAQ-II and MEAQ scores which were significant direct predictors of a likely PTSD diagnosis. The indirect effect of AAQ-II scores on the association between childhood trauma history and a likely PTSD diagnosis was significant, BCa CI [−0.0011, 0.0017]. MEAQ scores also showed a significant indirect effect on this relationship, BCa CI [0.0145, 0.0436]. After analyzing the total effect, the direct effect was reduced although still significant when the AAQ-II was analyzed as a mediator. In the second mediation model with MEAQ as the mediator, the direct effect became non-significant after analyzing the total effect. To ensure statistical differences would remain between the two measures of EA in the same model, these results were also computed through entering AAQ-II and MEAQ scores as mediating variables in a multiple mediation model. The partial mediating effects were replicated and thus, for more parsimonious comparison purposes, results of the single mediation models are solely reported. Results are displayed in Figure 3 and Figure 4.

## 4. Discussion

The aims of the present study were primarily to elucidate if measurement discrepancies exist in self-report measures of EA using the AAQ-II (the most widely used measure of EA) and the more recently validated MEAQ in their prediction of traumatic stress outcomes. It was hypothesized that both measures of EA would predict PTSS and problematic behaviors. However, it was further premised that the MEAQ, a measure that is conceptually more consistent with the operational definition of EA and with leverage over the AAQ-II in terms of psychometrics, would more accurately capture EA. Specifically, it was hypothesized that MEAQ scores would predict higher levels of PTSS beyond that predicted from the AAQ-II. 

AAQ-II and MEAQ scores were significant predictors of PTSS and problem behaviors as hypothesized and in the expected directions. While both measures evidenced partial mediating effects on the relationship between childhood trauma exposure and PTSS, when the measures were further used to predict a likely PTSD diagnosis, the MEAQ was a stronger predictor of a likely PTSD diagnosis, fully mediating the effects of childhood trauma history on a likely PTSD diagnosis as the outcome. While the AAQ-II did mediate this same relationship, the effect was smaller than the effect noted for the MEAQ. MEAQ scores showed stronger incremental contributions to a likely PTSD diagnosis, suggesting the MEAQ may be capturing a different construct than the AAQ-II. Interestingly, when the avoidance items were removed from PTSS/likely PTSD variables, results did not differ significantly.

Contemporary researchers posit that EA is a critical proximal risk factor, important to understanding the etiology and maintenance of trauma-related sequelae. Measurement discrepancies in EA were noted in the present study between the AAQ-II and MEAQ in regards to directly predicting a likely PTSD diagnosis. The AAQ-II appears to be a measure sensitive to predicting symptomatology in general as it predicted PTSS and mediated the effects of childhood trauma exposure on PTSS. However, when making group comparisons in the prediction of a likely PTSD diagnosis, the MEAQ demonstrated a stronger effect. Based on these initial findings, it is possible that the MEAQ may be a preferred self-report measure in capturing the EA construct as it relates to traumatic stress. MEAQ scores were also slightly stronger predictors of problematic behaviors. Those interested in understanding the relationship between EA and PTSS in both clinical and research contexts might consider using the MEAQ or the Brief Experiential Avoidance Questionnaire (BEAQ), a measure derived from the items of the MEAQ that consists of fewer items [34]. 

The AAQ-II is presently the most commonly used measure of EA in the psychological literature and trauma literature. To the authors’ knowledge, there exist no published methodological evaluations on the AAQ-II and MEAQ. However, these findings are consistent with another study that identified individual subscales of the MEAQ as predictors of PTSD symptomatology in a college sample [9]. Perhaps there are specific avoidance strategies (e.g., distraction and suppression) that are more widely used by trauma survivors that provide greater short-term relief from thoughts and memories and thus are more prevalent in trauma survivors. In this sense, the MEAQ which assesses content related to specific avoidance strategies may be more salient to predicting trauma while this may not be the case with other specified outcome variables. Traumatic stress may require more chronic patterns of EA that may not be necessarily to temporarily suppress or avoid general psychological distress. Future research in this area may help elucidate whether these effects are more specific to traumatic stress or generalize across psychological outcomes such as depression. 

The psychometric properties reported in the initial AAQ-II validation study [24], although acceptable, are less exemplary than that of the MEAQ [1]. In particular, the discriminant validity of the AAQ-II has been called into question. Many empirical studies include treatment outcome investigations of ACT that target and hope to reduce EA [35]. As such, use of a reliable self-report measure with good discriminant validity is especially integral. The present study provides initial evidence for the incremental effectiveness of the MEAQ beyond the AAQ-II in prediction of a likely PTSD diagnosis. However, future research will be needed to replicate these effects and extend the findings to a clinical sample. Of note, there are several limitations to the present study that warrant mention. It should be noted that the sample size of participants falling into the likely PTSD diagnosis category were relatively small in number (*n* = 78). Replicating these findings with a clinical sample will provide additional information and added power for making group comparisons between those with a likely PTSD diagnosis and those without. It should be noted that although a likely PTSD diagnosis category was explicated, no follow-up assessment in the form of clinical interviewing such as the Clinician Administered PTSD Scale was conducted. Therefore, the scope of confidence in these results is less certain. Additional research will be necessary to clarify a PTSD diagnosis through additional methodology beyond self-report screening tools. The present study was also limited in its use of cross-sectional research methodology. Repeated measures designs are necessary to assessing the temporal stability of EA, which are beyond the scope of this research project. It would be interesting to assess EA measurement differences temporally to provide additional support to these research hypotheses. Testing these hypotheses in a clinical sample will also be important to assessing measurement differences and may help substantiate the effects noted here, or provide new evidence for EA strategies in clinical samples. It is important to note these findings are preliminary and may not generalize beyond this convenience sample. Group differences were generated based on a dichotomy of unlikely versus likely PTSD diagnoses which could be strengthened through use of clinical interviewing to differentially diagnose PTSD and compare to a group of healthy controls. 

While there are some notable limitations to the present study, these initial findings in terms of EA measurement differences warrant further investigation and study. The MEAQ may be advantageous in terms of measuring specific EA strategies that clinicians and clients can target in clinical and research contexts. While self-report psychological screening tools rely on a degree of retrospective reporting that calls into question the veracity of said reporting, the MEAQ is one instrument that appears to provide enhanced construct validity more consistent with the operational definition of EA. That is, the MEAQ targets specific operant avoidance/escape strategies that individuals might use to alter contact with aversive private events. Conversely, the AAQ-II items are worded more ambiguously and do not clearly elucidate specific avoidance strategies (e.g., “I’m afraid of my feelings”). Supplementing research methodology with behavior analogue measures may also potentiate more effective measurement of this increasingly popular transdiagnostic process. 

## 5. Conclusions

Results of the present study provide evidence for measurement discrepancies in self-reported EA as assessed by the AAQ-II and MEAQ in their prediction of PTSS and problematic behaviors. Both measures were found to partially mediate the effect of childhood trauma exposure on problematic behaviors and PTSS. When PTSS was dichotomized into likely PTSD and unlikely PTSD, both the AAQ-II and MEAQ mediated the relationship between childhood trauma exposure and a likely PTSD diagnosis. While the direct effect still remained significant, it was more strongly reduced in the single mediation model estimated with the MEAQ as the mediating variable. These results, while cross-sectional and reported on a non-clinical sample, provide preliminary support that the MEAQ may possess advantages in terms of discriminant validity above and beyond the AAQ-II in predicting PTSS. 

## Figures and Tables

**Table 1:** Bivariate correlations for study variables.

Variables	1	2	3	4	*M(SD)*	*Range*
(1) MEAQ	-				226 (28.86)	114–332
(2) AAQ-II	0.61 **	-			22 (9.16)	7–47
(3) CTQ-SF	0.24 *	0.37 **	-		51 (15.39)	34–122
(4) PCL	0.39 **	0.62 **	0.40 **	-	39 (14.76)	16–76
(5) CMPB	0.29 **	0.32 **	0.28 **	0.37 **	115 (24.24)	72–201

Note: *N* = 226, *p < 0.05, **p < 0.01.
